# Correction: Investigating the functional and structural effect of non-synonymous single nucleotide polymorphisms in the cytotoxic T-lymphocyte antigen-4 gene: An *in-silico* study

**DOI:** 10.1371/journal.pone.0320345

**Published:** 2025-03-10

**Authors:** Md. Mostafa Kamal, Kazi Fahmida Haque Shantanu, Shamiha Tabassum Teeya, Md. Motiar Rahman, A. K. M. Munzurul Hasan, Douglas P. Chivers, Tanveer A. Wani, Atekah Hazzaa Alshammari, Mahesh Rachamalla, Francisco Carlos da Silva Junior, Md. Munnaf Hossen

There are errors in the Funding statement. The correct Funding statement is as follows: This work is funded by the NSERC Discovery Grant awarded to D.P.C. and a grant (Project Number RSP2025R357) from King Saud University, Riyadh, Saudi Arabia, for molecular dynamics simulations.
