# Supplementary material for: Investigating the functional and structural effect of non-synonymous single nucleotide polymorphisms in the cytotoxic T-lymphocyte antigen-4 gene: An in-silico study
Source: PLoS One. 2025 Jan 24;20(1):e0316465. doi: 10.1371/journal.pone.0316465 (PMC11759363; doi:10.1371/journal.pone.0316465)
Supplement: S1 Table — (DOCX) [file pone.0316465.s001.docx]

**S1 Table. KEGG Pathway enrichment analysis of *CTLA4* gene and its associate gene.**

| **Gene Set** | **Description** | **Ratio** | **P Value** | **FDR** |
| --- | --- | --- | --- | --- |
| hsa05235 | PD-L1 expression and PD-1 checkpoint pathway in cancer | 30.739 | 1.42E-09 | 5.00E-07 |
| hsa04660 | T cell receptor signaling pathway | 22.286 | 1.37E-08 | 2.4168E-06 |
| hsa04659 | Th17 cell differentiation | 21.423 | 2.25E-07 | 2.6449E-05 |
| hsa04514 | Cell adhesion molecules | 15.081 | 1.7974E-06 | 0.00015817 |
| hsa04658 | Th1 and Th2 cell differentiation | 21.225 | 2.8051E-06 | 0.00019748 |
| hsa05320 | Autoimmune thyroid disease | 29.965 | 8.0047E-06 | 0.00046961 |
| hsa04650 | Natural killer cell mediated cytotoxicity | 14.924 | 0.000015845 | 0.00079678 |
| hsa05416 | Viral myocarditis | 23.511 | 0.000021177 | 0.00093177 |
| hsa04917 | Prolactin signaling pathway | 21.831 | 0.000028436 | 0.0011122 |
| hsa05220 | Chronic myeloid leukemia | 20.934 | 0.000033584 | 0.0011822 |
