# Supplementary material for: Investigating the functional and structural effect of non-synonymous single nucleotide polymorphisms in the cytotoxic T-lymphocyte antigen-4 gene: An in-silico study
Source: PLoS One. 2025 Jan 24;20(1):e0316465. doi: 10.1371/journal.pone.0316465 (PMC11759363; doi:10.1371/journal.pone.0316465)
Supplement: Attachment — Submitted filename: PONE-D-24-35176_review_report_SN.pdf [file pone.0316465.s003.pdf]

Review Report

The manuscript Titled: “Investigating the functional and structural effect of non-synonymous single nucleotide polymorphisms in the cytotoxic T-lymphocyte antigen-4 gene: An in-silico study” presents an in-silico analysis of the effects of non-synonymous single nucleotide polymorphisms (nsSNPs) in the cytotoxic T-lymphocyte antigen-4 (CTLA4) gene. The authors used various bioinformatics tools, including SIFT, PolyPhen-2, PROVEAN, and SNAP, to identify deleterious nsSNPs. Additionally, they evaluated protein stability, structural changes, and interactions using tools like ConSurf, I-Mutant, and molecular dynamics simulations.

The manuscript is timely and covers an important topic, as SNPs in the CTLA4 gene have been implicated in immune regulation and several diseases, including cancer and autoimmune disorders. Using computational tools for analyzing genetic variants is appropriate for this study, and the results may serve as a foundation for further experimental work. However, there are several areas where the manuscript can be strengthened in terms of clarity, depth of analysis, and presentation of results.

Major Comments:

1. The study presents a comprehensive computational analysis of nsSNPs in the CTLA4 gene. The significance of this work lies in its potential to shed light on the pathogenicity of certain SNPs that might contribute to disease susceptibility. However, the manuscript could benefit from stronger contextualization of its results with reference to previous experimental findings. While the authors mention autoimmune diseases and cancer, a more detailed discussion on the clinical relevance of the identified nsSNPs would enhance the paper’s impact.

2. The manuscript employs a wide array of computational tools, which is commendable. However, some methodological details are either missing or could be clarified:

   Selection of SNPs: The criteria for selecting the 165 missense SNPs for further analysis are not fully explained. Were all missense variants analyzed, or was there a filtering process? Clarifying this would improve the transparency of the study.

   Protein Modeling: The molecular dynamics simulation section is interesting, but further details are needed regarding the parameters used in these simulations. Specifically, what were the time steps and temperature conditions? Were multiple simulations conducted for each variant to ensure reproducibility?

3. The results provide a solid foundation for understanding how specific nsSNPs affect the structure and function of the CTLA4 protein. However:

   - The explanation of molecular dynamics simulation results could be expanded. The authors report RMSD and RMSF values but do not provide a thorough interpretation of what these fluctuations imply in terms of biological function.
   - The protein-protein interaction analysis using GeneMANIA and STRING is intriguing but lacks depth. A more detailed discussion of the relevance of interactions with

proteins such as CD80 and CD86 in the context of immunological function would be beneficial.
- A visual representation of the molecular dynamics simulation results would significantly enhance the clarity of the findings.

4. The discussion section should place more emphasis on how the findings can inform future in vitro or in vivo studies. The authors briefly mention the need for experimental validation, but they do not elaborate on how their results could be applied in a practical setting, for example, by guiding targeted mutagenesis experiments or developing therapeutic interventions.

Minor Comments:

5. The manuscript is well-written overall, but there are several areas where the clarity of writing could be improved:
    - The abstract is dense and could benefit from more concise wording, particularly in the results section. Summarizing the key findings in a few clear sentences would make it more accessible to a broader audience.
    - In the results section, the use of terms such as “deleterious,” “probably damaging,” and “benign” are used without providing sufficient context for how these terms were determined by each computational tool. A short explanation in the methodology or results section about how these terms were defined would aid comprehension.
6. The manuscript contains a number of useful figures and tables. However, it would be helpful to:
    - Add more detailed captions for each figure and table. For example, in Table 4, providing more information on how the ΔΔG stability values were calculated and what these values mean in terms of biological relevance would enhance understanding.

    - Include a schematic that summarizes the workflow of the computational analyses performed. This would provide readers with a quick overview of the methodologies used.

7. The references are appropriate and up to date. However, the authors could strengthen the manuscript by citing more recent experimental studies that have validated nsSNPs in the CTLA4 gene, particularly in relation to cancer and autoimmune diseases.

Recommendations:

- Clarify the methodology, particularly the selection process for the SNPs analyzed and the details of the molecular dynamics simulations.
- Expand the discussion to include more implications for future experimental work and clinical relevance.
- Enhance the figures and tables by adding more detailed captions and including a workflow schematic.
- Improve the clarity of the writing, particularly in the abstract and results sections.

The manuscript presents valuable findings but requires minor revisions to improve clarity, detail, and depth of analysis. Once these revisions are made, the manuscript will strongly contribute to the field.
