# Supplementary material for: Investigating the functional and structural effect of non-synonymous single nucleotide polymorphisms in the cytotoxic T-lymphocyte antigen-4 gene: An in-silico study
Source: PLoS One. 2025 Jan 24;20(1):e0316465. doi: 10.1371/journal.pone.0316465 (PMC11759363; doi:10.1371/journal.pone.0316465)
Supplement: Attachment — Submitted filename: Response to Reviewers.docx [file pone.0316465.s004.docx]

**Reviewer Comments**

**Dear Reviewers**

Thank you so much for your comments. We have addressed all of your comments and revised our manuscript accordingly. All revisions are done under track change option. Responses of reviewer 1 are green highlighted, responses of reviewer 2 are yellow highlighted and responses of reviewer 3 are cyan colored highlighted in the revised manuscript.

**Reviewer #1**

Manuscript titled “Investigating the functional and structural effect of non-synonymous single nucleotide polymorphisms in the cytotoxic T-lymphocyte antigen-4 gene: An in-silico study’ presents good information about the nsSNPs in CTLA4. However, in my opinion following queries needs to be Responseed before accepting manuscript.

Major revision

1. dbSNP database (<https://www.ncbi.nlm.nih.gov/snp/>), respectively (accessed December, 2022) this is 1.5 years old data. What is the current status in this database for the CTLA4? Authors need to give current status also.

**Response:** Thank you for your comment. We have modified data and mentioned the current status of nsSNP of CTLA4 gene (accessed November, 2024).

2. A 100 ns MD simulation analysis is not sufficient for the prediction; at least triplicate analysis should be done for better predicted results.

**Response:** There has been done numerous functional and structural analysis along-side MD simulation.

3. Line 280“The structural stability of native and four mutant proteins were calculated RMSD and 281 RMSF and compare to evaluate structural and functional change due to the mutation of the 282-corresponding protein” correct English.

**Response:** We have revised accordingly.

4. Manuscript needs to be checked with reference to english properly. There are many grammatical errors.
**Response:** We have revised accordingly.

5. RMSD, RMSF, Rg values should be given in nm instead of A

**Response:** We have revised accordingly.

6. Secondary structure analysis should be done for wild type and mutant structures.

**Response:** We have already analyzed comparative modelling of wild type CTLA4 protein and their mutant structures.

7. PCA analysis should also be done to understand the structural impact of mutations.

**Response:** Thank you for your comment. We have added PCA analysis in the revised manuscript.

**Reviewer #2**

The manuscript Titled: “Investigating the functional and structural effect of non-synonymous single nucleotide polymorphisms in the cytotoxic T-lymphocyte antigen-4 gene: An in-silico study” presents an in-silico analysis of the effects of non-synonymous single nucleotide polymorphisms (nsSNPs) in the cytotoxic T-lymphocyte antigen-4 (CTLA4) gene. The authors used various bioinformatics tools, including SIFT, PolyPhen-2, PROVEAN, and SNAP, to identify deleterious nsSNPs. Additionally, they evaluated protein stability, structural changes, and interactions using tools like ConSurf, I-Mutant, and molecular dynamics simulations.

The manuscript is timely and covers an important topic, as SNPs in the CTLA4 gene have been implicated in immune regulation and several diseases, including cancer and autoimmune disorders. Using computational tools for analyzing genetic variants is appropriate for this study, and the results may serve as a foundation for further experimental work. However, there are several areas where the manuscript can be strengthened in terms of clarity, depth of analysis, and presentation of results.

**Major Comments:**

The study presents a comprehensive computational analysis of nsSNPs in the CTLA4 gene. The significance of this work lies in its potential to shed light on the pathogenicity of certain SNPs that might contribute to disease susceptibility. However, the manuscript could benefit from stronger contextualization of its results with reference to previous experimental findings. While the authors mention autoimmune diseases and cancer, a more detailed discussion on the clinical relevance of the identified nsSNPs would enhance the paper’s impact. The manuscript employs a wide array of computational tools, which is commendable. However, some methodological details are either missing or could be clarified:

Selection of SNPs: The criteria for selecting the 165 missense SNPs for further analysis are not fully explained. Were all missense variants analyzed, or was there a filtering process? Clarifying this would improve the transparency of the study.

**Response:** Thank you for your comment. We have retrieved the SNPs data from the dbSNP database which already mentioned in the methodology section. We have used missense variants as they are the disease-causing mutation (Zhang et al., 2012).

Protein Modeling: The molecular dynamics simulation section is interesting, but further details are needed regarding the parameters used in these simulations. Specifically, what were the time steps and temperature conditions? Were multiple simulations conducted for each variant to ensure reproducibility?
**Response:** All details regarding the parameters used in these simulations has already mentioned in this study, please see the methodology section in the revised manuscript.

The results provide a solid foundation for understanding how specific nsSNPs affect the structure and function of the CTLA4 protein. However:

The explanation of molecular dynamics simulation results could be expanded. The authors report RMSD and RMSF values but do not provide a thorough interpretation of what these fluctuations imply in terms of biological function. The protein-protein interaction analysis using GeneMANIA and STRING is intriguing but lacks depth. A more detailed discussion of the relevance of interactions with proteins such as CD80 and CD86 in the context of immunological function would be beneficial. A visual representation of the molecular dynamics simulation results would significantly enhance the clarity of the findings.

The discussion section should place more emphasis on how the findings can inform future in vitro or in vivo studies. The authors briefly mention the need for experimental validation, but they do not elaborate on how their results could be applied in a practical setting, for example, by guiding targeted mutagenesis experiments or developing therapeutic interventions.

**Response: --**

**Minor Comments:**

The manuscript is well-written overall, but there are several areas where the clarity of writing could be improved:

The abstract is dense and could benefit from more concise wording, particularly in the results section. Summarizing the key findings in a few clear sentences would make it more accessible to a broader audience.

**Response:** Thank you for your comment. We have revised accordingly.

In the results section, the use of terms such as “deleterious,” “probably damaging,” and “benign” are used without providing sufficient context for how these terms were determined by each computational tool. A short explanation in the methodology or results section about how these terms were defined would aid comprehension.

**Response:** Identification of deleterious nsSNPs in *CTLA4* gene, we have found the corresponding result from PolyPhen2 tools. We have already mentioned in the methodology section about this prediction and scoring system. Please see the revised manuscript.

The manuscript contains a number of useful figures and tables. However, it would be helpful to:
Add more detailed captions for each figure and table. For example, in Table 4, providing more information on how the ΔΔG stability values were calculated and what these values mean in terms of biological relevance would enhance understanding.

Include a schematic that summarizes the workflow of the computational analyses performed. This would provide readers with a quick overview of the methodologies used.

The references are appropriate and up to date. However, the authors could strengthen the manuscript by citing more recent experimental studies that have validated nsSNPs in the CTLA4 gene, particularly in relation to cancer and autoimmune diseases.

**Response:** Thank you for your comment. Please see the Figure 1, we have already mentioned the workflow. We have revised the manuscript accordingly.

**Recommendations:**
Clarify the methodology, particularly the selection process for the SNPs analyzed and the details of the molecular dynamic’s simulations.

**Response:** Thanks. Done.

Expand the discussion to include more implications for future experimental work and clinical relevance.

**Response:** Thanks. Done

Enhance the figures and tables by adding more detailed captions and including a workflow schematic.

**Response:** We have revised the manuscript accordingly.

Improve the clarity of the writing, particularly in the abstract and results sections.
**Response:** We have revised the manuscript accordingly.

The manuscript presents valuable findings but requires minor revisions to improve clarity, detail, and depth of analysis. Once these revisions are made, the manuscript will strongly contribute to the field.

**Response:** Thank you.

Reviewer #3

Title: Investigating the functional and structural effect of non-synonymous single nucleotide polymorphisms in the cytotoxic T-lymphocyte antigen-4 gene: An in-silico study

1. In section 3.1, Accession number of the sequence is missing, please provide it, in order to verify the result?

**Response:** Thanks for your comments. We have revised accordingly and added Id. Please see page line in the revised manuscript.

2. In line 219, You mention 5 nsSNPs for SNAP and 4 nsSNPs for PROVEAN, but it might be useful to explain why different numbers of nsSNPs were used for these analyses?

**Response:** We have corrected this number in the revised manuscript.

3. In line 227, Avoid Vague Phrasing: Instead of “notable consistency,” I used “strong concordance” to make the statement more specific.

**Response:** Done.

4. Use the correct symbol for free energy change (ΔΔG instead of DDG) to maintain consistency with standard scientific terminology.

**Response:** We have revised accordingly.

5. In Line 238 and 239, specify what a conservation score of 4, 5, and 9 indicates in terms of evolutionary conservation, as readers might not be familiar with the ConSurf scoring system.

**Response:** Thank you for your comment. We have added adequate data in the revised manuscript and It has already been mentioned in figure 2B.

6. Please correct the caption of Table 3.

**Response:** We have revised accordingly.

7. In Line 246, can you please focus that why the size of amino acid residues is responsible for protein’s function disruption?

**Response:** We have revised accordingly. Please see page line in the revised manuscript.

8. Alanine residue is generally an accepted single residue first choice for mutation, can you try to mutate the key residues to Alanine here and study the result?

**Response:** Thanks for your comments.

9. Section 3.8 should be improved by explaining how protein interactions can impact network function.

**Response:** Thanks for your comment. We have revised accordingly.

10. In Section 3.9, since you simulated all the systems for 100 ns, could you incorporate principal component analysis (PCA) to examine the atomic movements, particularly at the mutated residue sites?

**Response:** We have added PC analysis in the revised manuscript.

11. In the Materials and Methods section, while you mention that molecular dynamics simulations were performed using the Desmond v6.3 program, there is no information provided regarding the number of steps for energy minimization, position restraints, or equilibration. Please include these details.

**Response:** We have already added all information regarding molecular dynamics simulation. (Protein structure system minimized using a natural time and pressure (NPT) ensemble at a constant pressure of 101325 Pascal’s and a temperature of 300 K).

12. Since you simulated the systems in a water medium, you should also include solvent accessible surface area (SASA) analysis to examine the role of water around the mutated regions and compare it with the wild type. This is particularly relevant since you previously mentioned that the mutations were based on the shape of the amino acid residues.

**Response:** We have added PC analysis in the revised manuscript as SASA is not possible for this corresponding study for limitation of facilities.

13. How many simulation repeats did you performed?

**Response:** Thanks for your comments.

14. Please correct the typographical and grammatical errors throughout the manuscript to improve its readability and clarity.

**Response:** Thanks for your comments.

15. The plot in Figure 7 is unclear. Please add the average plot of hydrogen bonds for each system to make the data for each system more distinguishable.

**Response:** We have provided the supplementary data of figure 7 in the revised manuscript. Please see the supplementary table S2.
